# It’s a Man’s World: A Qualitative Study of Gender and Sexuality amongst Australian Gay Men

**DOI:** 10.3390/ijerph19042092

**Published:** 2022-02-14

**Authors:** Jack Thepsourinthone, Tinashe Dune, Pranee Liamputtong, Amit Arora

**Affiliations:** 1 School of Health Sciences, Western Sydney University, Penrith, NSW 2751, Australia; t.dune@westernsydney.edu.au (T.D.); a.arora@westernsydney.edu.au (A.A.); 2 Translational Health Research Institute, Campbelltown Campus, Western Sydney University, Locked Bag 1797, Penrith, NSW 2751, Australia; 3 College of Health Sciences, VinUniversity, Gia Lam District, Hanoi 100000, Vietnam; pranee.l@vinuni.edu.vn; 4 Health Equity Laboratory, Adelaide, NSW 2560, Australia; 5 Oral Health Services, Sydney Dental Hospital, Sydney Local Health District, NSW Health, Sydney, NSW 2010, Australia; 6 Discipline of Child and Adolescent Health, The Children’s Hospital at Westmead Clinical School, Faculty of Medicine and Health, The University of Sydney, Parramatta, NSW 2145, Australia

**Keywords:** internalized homonegativity, homonegativity, masculinity, LGBT, gender norms

## Abstract

Currently, research explicitly examining masculinity and internalized homonegativity is sparse, and even sparser studies are those using qualitative methods. To address this, this study aims to explore: how gender norms are constructed and experienced amongst gay men; and how gender and sexual identity are experienced in relation to masculine norms amongst gay men. A sample of 32 self-identified gay men aged 22–72 years (*M* = 34.34, *SD* = 12.94) participated in an online semi-structured interview on masculinity and homosexuality. The study used Zoom to facilitate the online interviews as it offered privacy, accessibility, ease of use, and voice recording, among other benefits. Thematic analyses revealed gay men’s understandings of masculinity, femininity, and sources of pressure to conform. Furthermore, gay men emphasize the conflict experienced between heteronormative gender and sexuality norms, which highlights the term homosexual male as an oxymoron.

## 1. Introduction

Gender norms are a pervasive social structure that provide prescriptions that both guide and constrain an individual’s behavior (e.g., men ought to be strong) [1,2]. They operate under a framework in which behavior is gendered, generalized, strictly scripted, and socially governed in order to avoid derision [3]. Bradley [4] notes that heteronormative masculinity is defined by the exclusion and oppression of its outgroup actors—women and gay men—who threaten its very essence. Although women may resist hegemonic femininity and adopt more masculine traits (e.g., butch, tomboy) without comparatively much hostility, men who adopt more feminine traits often experience social backlash from strangers, friends, and even family members—notably fathers [4]. Despite current social trends, social dichotomization between femininity and masculinity continues to exist [5,6]. This suggests a rigidity in male gender norms, as compared to female gender norms, whereby social trends favor divergences from traditional gender norms for females but not for males. One such example includes the general social acceptance of women wearing either pants or dresses while men only wear pants. In this paper, we therefore draw particular attention to masculine–heteronormative gender norms—specifically, what is perceived to be masculine, how non-heteronormative men relate to masculinity, and its attributing role in the lives of non-heteronormative men.

### 1.1. Masculinity and Homosexuality: Literature Review

The current literature maintains that heteronormative masculinities depict gay men as being more feminine than their heterosexual counterparts and affect perceptions of their gender and sexual identity [7,8,9]. Phrases such as “that is so gay” or “no homo” are often used as a form of social regulation to deter unscripted expressions of masculinity [10]. However, not only do phrases like these reflect society’s perceptions of homosexuality, but they also reflect heterosexist ideals. As a consequence of these heterosexist ideals, gay men often experience negative attitudes towards their own sexuality—internalized homonegativity [11]. Internalized homonegativity has been noted to relate to depression, poor wellbeing, sexual discrimination, shame, body dissatisfaction, eating disorders, and suicidal ideation, and results in more extreme and unbearable states of mind in men than women [12,13,14,15,16,17]. Phrases such as “I am a man, therefore I may not love a man” [18] and “you can’t be a man and be gay” [7] are common concepts that gay men are regularly confronted with. Additionally, anti-effeminacy and homophobic sentiment has been argued to be cyclically perpetuated by victims of gender/sexuality harassment [19]. Minority stress theory argues that homonegative and heterosexist social environments contribute to gender and sexuality diverse individuals’ experiences of chronic stress [20]. As such, it is imperative that the role of gender norms (specifically, masculinity) on non-heteronormative individuals’ experiences of gender and sexual identity be addressed to alleviate minority stress, improve mental health, wellbeing, and quality of life, and reduce heteronormative pressures.

Among gay men, homosexual masculinity is often referred to as “straight-acting” masculinity and is argued to be an emulation of heteronormative masculinity—and, arguably, heterosexuality [21,22]. The term straight-acting bears heterosexist overtones, and using this identifier in place of masculine suggests an ideology that masculinity is a state of being exclusive to heterosexuality. HWhile heterosexual masculinity is perceived as an authentic and natural gender performance, whereas homosexual masculinity is perceived as a costumed drama [23]; “homosexual men are more like women…Even the most masculine gay man is a bit sissy” [24]. The social construction of masculinity, therefore, generates a reified oxymoron out of the phrases “homosexual male” and “gay man”, whereby both terms are perceived as direct oppositions of each other.

The strain gay men may experience in their efforts to be as heteronormatively masculine as possible, despite their homosexuality, is perpetuated by prejudice and discrimination in all areas of life that serve to govern socially acceptable expressions of masculinity. For example, gay men who are overly concerned with gender norms and masculine body ideals are argued to be compensating for their feelings of internalized homonegativity and inferiority [8,25,26,27]. Furthermore, individuals who have experienced harassment due to childhood gender non-conformity are more likely to experience later adult life body shame and bear anti-effeminacy prejudice towards others [19,28]. This is evident in discriminative social practices in classifieds and dating applications that exclude effeminate men [25,29,30,31]. As a result, gay men who have internalized heteronormative masculinity and the strict rules therein participate in policing other gay men, as well as themselves through compensatory behavior, as a means of minimizing gay men’s effeminacy stereotypes [19]. It can be further argued that the discrimination between straight-acting and effeminate gay men, particularly within personal advertisements, normalizes, and even glorifies, the divisive social practice. These dynamics thus perpetuate heteronormative masculinity, (hyper)masculine gender norms, and further contribute to gender-related strain and internalized homonegativity.

### 1.2. The Australian Social Environment

The social environment of gender and sexuality diverse groups, within a Western context, is rapidly evolving [32]. It is only within the past century that more positive attitudes have emerged. In 1957, psychologist Evelyn Hooker challenged the mainstream view of homosexuality being a disorder [33]. In 1973, the Diagnostic Manual of Mental Disorders no longer classed homosexuality as a disorder. This was subsequently followed by changes within the World Health Organization’s International Classification of Diseases in 1990 [34]. However, progress within Australia has been slow. The campaign to decriminalize same-sex sexual conduct in Australia began in the 1970s, but it was not until 1997 that it became legal in all Australian states [35]. Furthermore, it was only within the last five years—December 2017—that Australia passed the Marriage Amendment (Definition and Religious Freedoms) Act 2017, which legalized marriage between same-sex couples [36].

Prior to this, discrimination against gender and sexuality diverse groups was common, with arguments of gay and lesbian relationships being unnatural [36]. Similarly, the period leading up to the amendment saw a rise in homophobic and transphobic harassment and assault [37]. Sentiments such as “I was really scared, I don’t feel as safe as I used to” was common among gender and sexuality-diverse individuals [37].

### 1.3. Theoretical Framework

As this paper explores the role and relationship between factors that influence how masculinity is internalized and experienced in line with (non)heteronormative gender norms, a holistic theoretical lens is needed to interpret the data collected. We therefore engage with the principles of socio-ecological theory [38,39]. Doing so will allow for a multidimensional view of the interactions and relationships between a wide range of factors within a person’s environment. Socio-ecological theory supports this as it helps to identify constructs, interactions, and experiences between an individual and various levels of their environment. Flanders et al. [40], for example, argued that the use of the framework allowed for an explicit analysis of the gender socialization process through each level of the individual’s environment. It helps to provide additional and holistic insights into the social intricacies and dimensions of gender and sexuality, including how they shape and are shaped by the individual and their environment, which other studies tend to overlook.

Socio-ecological theory emphasizes the agency of both the individual and the influence of their environment, as each shapes and is shaped by each other [38,39]. This includes both informal and formal environments such as:

- The microsystem—family and close social networks;
- The mesosystem—major settings (e.g., school, church, work);
- The exosystem—other social structures that, although do not contain the individual, encompass their immediate setting;
- The macrosystem—broader social structures and ideologies.

**Figure 1:**
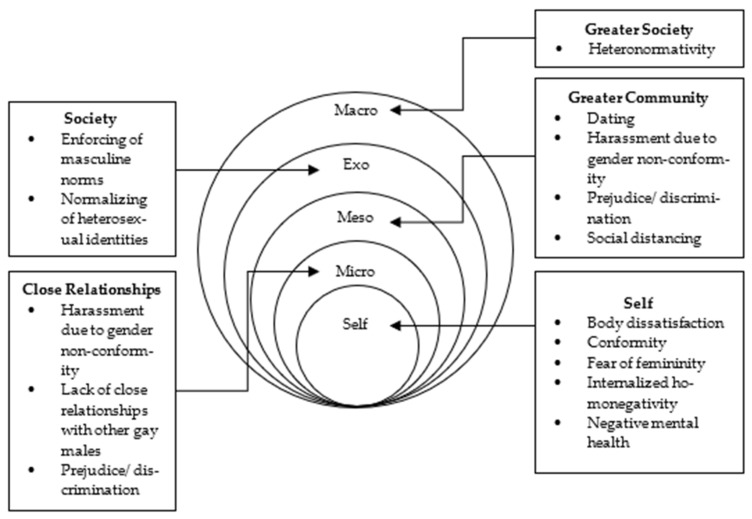
Socio-ecological map of an Australian gay man [44].

Importantly, social structures such as gender norms continue to exist and take shape according to those who use them [41]. Based on a synthesis of literature, Figure 1 depicts a preliminary conceptual model of the typical ecological environment for a gay man and highlights various areas heteronormative masculinity pervades and exerts an influence. Beginning from the macro level of a gay man’s social ecology, heteronormative masculinity permeates the psychology of how they perceive themselves, others, and the world and extends to the micro- and meso-systems that enforce heteronormative gender and sexual identities through interactions with friends and family members (microsystem), as well as strangers and colleagues (mesosystem) [7,19,25,29,30,31,42]. Moreover, the interactions between the individual and their micro-, meso-, and exo- systems arguably contribute further to the issues presented [8,19,43]. It can, therefore, be argued that the role of heteronormative masculinity on a gay man’s life cannot be examined in isolation but, rather, as a complete system of factors, each contributing to the manufacturing of internalized homonegativity. The present study seeks to explore and identify the factors within each level of the socioecological model that influences gay men’s identity, expressions, and experiences of masculinity and gender norms in the context of (non)heteronormativity.

### 1.4. Study

Currently, research explicitly examining masculinity and internalized homonegativity is sparse [16,24,45] and even sparser are studies examining internalized homonegativity qualitatively [13]. This paper is based on a larger body of work titled “It’s a Man’s World”, which explores masculinity and internalized homonegativity amongst gay men. The study, therefore, aims to qualitatively explore this under-examined area and develop a stronger understanding of the relationship between masculinity and internalized homonegativity within a sample of Australian gay men. The research aims to explore:

1. How gender norms are constructed and experienced amongst gay men; and
2. How gender and sexual identity is experienced in relation to masculine norms amongst gay men.

It is expected that the findings will contribute to the identification of the underlying issues surrounding internalized homonegativity (e.g., gender norms) and gaps for further research in the area.

## 2. Materials and Methods

### 2.1. Research Design and Instruments

Few studies have examined masculinity and internalized homonegativity qualitatively [13]. This study, therefore, intended to use an under-examined methodology within the field. With considerations of previous studies [7], semi-structured interviews were utilized. The interview focused on men’s experiences (or lack thereof) of internalized homonegativity, the factors that contribute to their experiences, and the impact it may have on their health and wellbeing. As such, topics for discussion were centered on: experiences of childhood harassment for gender non-conformity, notions of homosexuality as feminine, pressure to be straight-acting/masculine, reactions to gender non-conformity (e.g., anti-femme), and negative feelings about being gay. Table 1 contains the interview guide used. The research design and ethical considerations were initially reviewed by a panel of three experts in the field, as well as receiving further review and approval by the Western Sydney University Human Research Ethics Committee (Approval No.: H12044).

All interviews were conducted via Zoom version 3.5.4 (Zoom Video Communications, San Jose, CA, 2017). A close-ended self-administered demographics questionnaire was also utilized to ascertain participant’s background information—age, gender, ethnicity, religion, post code, and from what device were they accessing in order to participate (e.g., laptop).

#### Using Zoom for Data Collection

The online environment is an invaluable resource in research, as it offers a safe and inclusive space, allowing for researchers to make visible that which is difficult to study or non-existent in traditional environments [46,47]. As such, we used Zoom, an online conferencing program, to collect the data. This software tool was utilized for two reasons: (a) it allowed for the interview to be recorded without the aid of additional software or equipment, and (b) it offered privacy in that it did not require the participant or researcher to add each other to their contacts—a common feature in online conferencing and social media. This allowed for the researcher to reach out and include underrepresented samples, geographically and/or socially isolated individuals, individuals who are unable to or prefer not to attend in person [48,49,50]. Gay men, for example, may not wish for their identities to be disclosed, and online environments, therefore, allow for such populations to participate in research with lower risks to their anonymity [48]. Furthermore, Zoom is argued to be more favorable among both participants and researchers over face-to-face, telephone, and other videoconferencing technologies when conducting interviews [51]. Other benefits of this particular software include convenience, ease of use, cost effectiveness, data management, interactivity, security, unique features such as video recording, and its ability to facilitate personal connections between users [51]. In this study, a personal computer with a reliable internet connection, web camera, and Zoom installed was used to facilitate the interview.

### 2.2. Participants

Participants from the It’s a Man’s World study were initially recruited via advertisements through lesbian, gay, bisexual, transgender, and/or intersex (LGBTI) networks (e.g., LGBTI Alliance of Australia, Queensland Aids Council), social media (e.g., Facebook, Twitter, Instagram), dating applications (e.g., Grindr), flyers placed across Western Sydney University campuses, and word of mouth. The researcher’s contact details were included within the advertisements in order for individuals to express interest in the study. Participants from the It’s a Man’s World study were given the option to also express interest in the current research and provide their contact details. A pool of 253 individuals self-identifying as gay men expressed interest in the current study after participating in the initial It’s a Man’s World study. All individuals were contacted through email after, and of these, only 32 individuals followed up by arranging an interview. No interviews were cancelled or turned down, and no participants withdrew from the study.

Between March and July 2017, and months prior to the legalization of same-sex marriage in Australia, a sample of 32 self-identified gay men aged 22–72 years (*M* = 34.34, *SD* = 12.94, median = 30) living in Australia (NSW = 90.63%, QLD = 3.13%, VIC = 3.13%, WA = 3.13%) participated in online interviews focusing on masculinity and homosexuality—this time period was marked with high contention and discussion around LGBTI issues. Among the sample, 3.13% identified as Aboriginal, 6.25% as East Asian, 6.25% as Southeast Asian, and the remainder as Caucasian (85.38%). Additionally, most of the sample identified with no religion (68.75%), followed by those identifying as Christian (18.75%), Buddhist (6.25%), and other (6.25%). Gay men, compared to lesbian women, are argued to be most adversely affected by heteronormative constructions of masculinity and femininity and are more prone to resultant health and wellbeing complications [8,16,52]. As such, the study’s aim and scope focused solely on gay men, and individuals of other genders and sexualities were excluded (e.g., transgender, bisexual, etc.). Each interviewee received a AUD 30 digital gift card as compensation for their time and inconvenience.

### 2.3. Procedure

Following initial contact, JT forwarded details of the study to the participants, including a participant information sheet, a participant consent form, instructions on how to install and use the Zoom program, and the time of the scheduled interview. On the day of the interview, participants were required to click on the link included in the email correspondence which automatically runs the Zoom program with the appropriate conference room for the present study. Audio recording was then enabled on Zoom once consent had been given, and JT commenced a semi-structured interview using the interview questions as a guide.

### 2.4. Analysis

Following data collection, interviews were transcribed verbatim and inserted into *Quirkos*. *Quirkos* is a visually intuitive data management software that assists researchers in the coding and analyses of qualitative data [53]. *Quirkos* assisted in organizing topical responses and emergent substantive categories. Thematic analysis method was used to analyze the data. It was conducted by ascertaining codes, patterns, and substantive categories within participants’ accounts in relation to the study’s aims [54]. Coding was conducted by JT, and emerging themes were discussed by all authors (JT, TD, PL, and AA).

## 3. Results

Pseudonyms were assigned to participants where direct quotes were used to maintain participant anonymity. Four distinct themes emerged from the gay men’s stories: Understanding Masculinity, Understanding Femininity, Gay Men and Gender Expression, and Sources of Pressure. The themes of Understanding Masculinity and Understanding Femininity relate to gay men’s perceptions and understanding of masculine and feminine gender norms, respectively. Similarly, the theme of Gay Men and Gender Expression relates to how gay men relate to, perform, and express gender and gender norms. Furthermore, Sources of Pressure relates to sources of pressure and expectation of performing/expressing gender norms.

### 3.1. Understanding Masculinity

When asked about masculinity, participants described masculinity using a range of characteristics and discussed it in relation to several systems (e.g., micro-, meso-, exo-, and macro-systems). Physical characteristics were one of which participants commonly referred to. For instance, muscularity, fitness, body weight, body height, deep voice, and body hair were quite common. Body weight was suggested to equate to masculinity; “I was never really a masculine person anyway. I’m as skinny as a stick… It’s actually interesting that I just conflated masculinity with body types” (Ernest, 26).

Other participants also described masculinity using lifestyle choices and behaviors. These included maintaining an active lifestyle, going to the gym, owning sports cars, trucks, or utes (utility vehicles), skills (e.g., repairing), ways of dressing, and even favored music genres. For instance, one participant characterized masculinity as “someone that goes to the gym and drives a big beefy car” (Anthony, 23) while another stated “being masculine is driving big trucks or being manly, rugged, and knowing how to fix things around the home” (Harry, 32).

Certain ways of communicating, expressing, interacting, and thinking were also associated with masculinity. Participants tended to describe masculinity as being less emotional, less affectionate, proud, egotistic, narrow-minded, misogynistic, and even homophobic. Generally, participants commonly described masculinity as restrictive. For instance, “not talking about your emotions… maybe suppressing the desire to just act a little bit femme and a bit softer sometimes” (Finn, 33) and “a limited range of topics that you can speak about with other men or even in general, in public, or in social situations” (Aaron, 24). Masculinity was also described as “having a really big sense of pride and ego” (Nathan, 26) and to place a limit to their behaviors: “I had a lot of trouble with the idea of specific types of music that I felt like I shouldn’t be listening to because of a need to be masculine” (Aaron, 24).

### 3.2. Understanding Femininity

Similarly, participants tended to describe femininity using lifestyle choices and behaviors, for instance, mannerisms, interest in fashion/shopping, ways of dressing, and certain behaviors. Largely, femininity was associated with femaleness and behaving “girly or acting like a girl” (Ernest, 26), camp, or flamboyant. One such example includes “wearing maybe bright colors or tight clothing or revealing clothing” (Melvin, 30). Gossiping was another behavior that participants described as feminine.

In terms of communicating, expressing, interacting, and thinking, femininity was commonly described as emotional, open, freeing, less serious, and empathetic. One participant expresses the positive impact femininity has on their gender expression; “For me it feels more free. I feel like I can be expressive. I can throw my hands around and I can dress in crazy ways. I can really be emotional. I can just really react in a strong emotional way to things. If someone tells me something I can be like ‘My god’. You can just really express yourself and you don’t have that limit on expressing yourself” (Nathan, 26).

### 3.3. Gay Men and Gender Expression

#### 3.3.1. Enacting Feminine Gender Norms

When asked about how gay men relate to the constructs of masculinity and femininity, a mix of responses were given. Some participants described gay men as being typically feminine and others as typically masculine. In the case of the former, participants expressed notions of femininity as a norm for gay men; “I think it’s also that gay men feel less inclined to have to live up to masculine ideas” (Aaron, 24) and “I thought maybe being feminine actually becomes a way for gay men to fit into a community and find a community and if you don’t fit into that maybe it’s a bit isolating” (Nathan, 26). This norm for gay men to adhere to feminine norms is further explained by one participant’s struggles to be welcomed by the gay community in their city; “You might see the whole scene and realize I don’t really fit in here and feel kind of crap. If you’re not popular or look a certain way or fit into that kind of homogenous 2010 kind of gay scene, then you might feel a bit shit about yourself so you reshape yourself to look and act in a certain way so that you fit in” (Finn, 33).

From the interviews, participants expressed experiencing prescriptions and expectations to enter more female-dominated careers, speak, behave, and dress a certain way, as well as participate in recreational activities deemed appropriate and congruent with their sexuality. Gay men are often compared to or described as being more like women: “People are more likely to make derogatory comments or call you a girl or call you one of the girls or assume you relate better to women than to men or assume you’re bad at sports. I think it also shows in subtle ways to being left out of certain things because you’re not as highly valued” (Melvin, 30). Another participant expressed, “A lot of the time, people’s response whether they be gay or straight… might be that the effeminate straight guy is clearly a closet case… whereas with femme gay guys, it’s just one of those things where people are like ‘Yeah, that is just how gay guys act’” (Finn, 33). Participants also expressed concern over the blurred line between gender identity and sexual identity: “When I introduce myself to some people, they go ‘I never would have thought you were gay’ or ‘Are you sure?’… Am I too masculine to be gay?” (Cooper, 26).

#### 3.3.2. Enacting Masculine Gender Norms

On the other hand, however, some participants expressed pressure to behave masculine, as well as negative social cues when behaving effeminately. For instance, one participant compared anti-effeminacy reactions in relation to straight and gay men: “I feel like with straight people it’s not so much a thing…Some guys might find it a little bit confusing and off-putting but I feel like gay guys are kind of worse about it” (Finn, 33). Similarly, another participant argued that “there is a toxic nature around what is seen as attractive in the gay male world and I feel that it usually favors fitness and muscles more” (Cooper, 26) and “to act like a woman is somehow a negative thing”.

Similarly, masculinity has been described to bear weight in the hierarchy of gay men and social status. For instance, one participant stated: “These buff, gym going, bearded hair dudes are sort of dominating the space and going ‘Well you’re actually too feminine. I’m masculine, so I’m more important than you. I’m more valid than you are’. It’s interesting to navigate that, because the way that society sort of pushes these more masculine sort of men, I feel they’re not as gay, if that also makes sense” (Cooper, 26). While others expressed “I think masculinity is viewed as a benchmark in which people are judged. The more masculine someone is, the better they are” (Ernest, 26) and “The more masculine you are, the higher up you are” (Melvin, 30). This form of ostracization and discrimination is described as being more common than other overt forms of discrimination: “Rather than pointing and calling names… now we simply exclude them. You’re not behaving straight enough for me and therefore I’m going to exclude you out and I only want to meet straight acting men. It’s not portrayed as a personal preference; it’s portrayed as somehow being better than the alternative” (Tyler, 51).

In examining the discrepancy between expectations of various gender norms among sub-communities/groups, the phenomena were described as self-perpetuating, cyclical, and, at times, further reinforcing and exaggerating such norms. One participant noted: “I think that sometimes we reinforce our own gender stereotypes by finding the other people that make us feel comfortable and then we build on that by bouncing off each other, and I think that happens for both straight acting and I think that happens for effeminate men as well. They’ll find themselves a group of people who behave in the same way. Having done so, they then feel more comfortable in behaving even more extreme in whatever behavior it is, be it spitting on the ground or be it having short shorts and jumping around and squealing. We support our own perceptions by finding people who make those perceptions comfortable” (Tyler, 51).

#### 3.3.3. Public and Outward Presentation

Others also discussed the stigmatization of male effeminacy, the fear of publicly presenting femme, and the use of masculinity as a defense mechanism. For instance, one participant described the use of heterosexual/masculine self-presentation as a form of defense mechanism: “I could be dressed in just a straight passing outfit, walking like normal…and I’ll still be afraid that they will perceive that I’m gay or not super masculine” (Aaron, 24). Similarly, another participant asserted that “if there was some kind of bikie who walked in and looked as scary as all hell, then I’d probably just try to be as normal as possible” (Ernest, 26). Similarly, “I think it’s wanting to conform… there’s a reason why men would be in the closet, it’s because they think it’s something that’s undesirable, to be a gay man” (Phillip, 23). 

Outward presentation of femininity is often described as an indicator of gayness. One participant expressed; “People are surprised when they find out that you’re gay or whatever and they’re like ‘I didn’t realize you were gay. Wow’, like that whole surprise, which underlying that is the implication that people should be readily identifiable based on how they act or whatever. I mean how are you going to find out that I’m gay unless I tell you that I like to bang dudes” (Finn, 33). Similarly, another participant expressed: “I was a lot more flamboyant when I was younger and it was actually brought to my attention via a couple of people saying, ‘People think you are gay’. They didn’t say, ‘You are gay and that’s okay’. They said, ‘It doesn’t matter if you are but people think you are’” (Phillip, 23). This is further depicted by one participant’s anecdote; “I think a lot of people get quite annoyed by flamboyancy and public displays of affection and, ‘I’m fine with what you do in your own bedroom kind of thing but don’t let me have to see it or know about it’” (Phillip, 23). These indicators of gayness may even include the most subtle of social nuances/physical details, for example, “I feel as if though the policing is of gender and of demonstration or performance of gender, but there’s an undertone there. It’s dad saying ‘Cut your fingernails because they’re too long’, but what he’s not saying in words, reading between the lines, he’s still saying ‘Stop looking so gay’” (Cooper, 26).

### 3.4. Sources of Pressure

When asked about sources of pressure, expectation, and inheritance of gendered norms, gay men described a plethora of sources stemming from all four systems (i.e., macro-, exo-, meso-, and micro-systems). For instance, one participant highlights the pervasive nature of gender norms through one’s society: “As much as I detest society says we’re supposed to think and feel, I do believe that does transcend into what I do, think, and feel sometimes, no matter how much I resist it” (Harry, 32). Other sources included: fathers, male figures, mothers, religion, school, authority figures, television, and movies. Furthermore, external pressures are described to permeate through to one’s own cognition: “I wouldn’t be able to drive a bright pink Barina down the main street of [city]. I couldn’t have the guts to do that, as ridiculous as that is. It’s a car. But I still, in my head, do feel the pressure of society saying I still have to act a certain way or do a certain thing or behave in a way and a manner in context rather than doing exactly what I want to do” (Harry, 32). One participant describes the lifelong process of gender socialization: “We grow up in a world where from the minute a child is born, they’re told that they have to behave in a certain way, and if they step outside of those rules, they’re punished. From such an early age, they’re told non-normative genders, non-normative sexualities are bad things” (Ernest, 26).

Additionally, participants often emphasized that the impact of such agents could occur through either direct or indirect interactions with the individual. For instance, one participant highlighted that “You don’t even have to have it directly put on you, you just have to hear it and be a witness to it happening to other people to start learning that that is the way things are” (Aaron, 24, Caucasian). Similarly, another participant emphasized the unspoken nature of gendered norms and the expectations of how (gay) men ought to be; “There’s an awful lot that we don’t appreciate that children pick up on as an unspoken” (Tyler, 51).

## 4. Discussion

**Figure 2:**
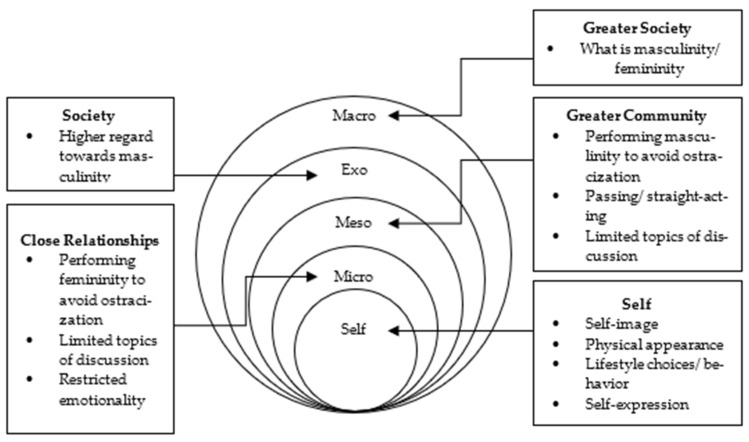
Socio-ecological map of an Australian gay man within the present study.

The paper has identified areas of gay men’s social environments that influence their sense of gender, sexuality, experiences of gender norms, and the impact it may have on their health and wellbeing. Figure 2 provides an overview of the results in accordance with their respective level on the socio-ecological model.

Gay men in the present study tended to emphasize physical characteristics and certain lifestyles (e.g., tradespersons) when describing masculinity. Like muscularity, a larger body weight bears with it a larger physical presence. This is consistent with traditional notions of masculinity, which include strength and dominance [1]. Such descriptors depict a physically strong, proficient, and skillful individual—typical of a blue-collar worker. Additionally, these characteristics may also be argued to depict activity as opposed to passivity—commonly attributed to masculinity [55] and dominance, both physically and socially/symbolically (e.g., owning a ute/truck may suggest manual/physically demanding occupations while owning a sports car may suggest wealth/affluence). Social structures existent in an individual’s exo- and macro-systems (e.g., masculinity) are argued to both shape, as well as are shaped by, individuals [41]. Within the previous examples, participants described instances whereby certain lifestyles influence what is perceived as masculine. However, the reverse was also be observed in a participant’s anecdote whereby constructs in one’s macrosystem impedes on one’s personal choices (i.e., choice of leisure activity).

Results also revealed ways in which masculine norms impeded on gay men’s ways of communicating, expressing, and thinking. This is consistent with extant literature highlighting masculine norms to include restrictive emotionality, restrictive affectionate behavior between men, success, power, competition, and primacy over women [56,57]. This demonstrates how masculinity influences how individuals interact with others within their micro- and meso-systems—specifically, it depicts an impediment on one’s relationships with others.

Within one’s exosystem, there exists the stereotype that gay men are more effeminate [7,9]. This stereotype affects how gay men perceive and relate to their own gender and sexual identities. Gay men within the present study revealed pressure and expectation to abide by feminine norms lest they be ostracized. This suggests that gender norms are reversed for gay men. While masculine gender norms may play a role in impacting gay men’s gender identity, feminine gender norms exert a pressure onto gay men due to their sexual identity. That is, masculine norms exert an influence over gay men due to their identity as a man while feminine norms exert an influence over gay men due to their identity as being gay. As such, gay men experience a discrepancy between their gender and sexual identities and feel compelled to perform their gender as what is typically viewed as feminine/gay. This force from agents within one’s meso- and micro-systems is argued to impact how gay men perceive themselves, as well as how they interact with others in their environment.

The present study’s results also revealed strain exerted by other gay men through the ostracization of feminine gay men and the veneration of masculine gay men. This is consistent with current literature, which emphasizes the discriminative social practices which exclude effeminate gay men [22,25,29,30,31]. These findings demonstrate the negative effect structures within an individual’s macro- (masculinity) and exo-systems (heteronormative gender norms) have on the individual and their interactions with their micro- and meso-systems through day-to-day social interactions, dating, and even how they present themselves to others. This hierarchy and valuation of masculinity is often described as part of straight-acting culture whereby passing as heterosexual was coveted. Additionally, this ritualized legitimization of gender among gay men demonstrates the influence agents within the meso- and micro-systems have in the construction of broader social structures [41].

Extant literature highlights the pressures received from friends and family members (e.g., fathers) in their microsystem, as well as strangers and colleagues within their mesosystem [4,25,29,30,31]. Gay men, hence, experience pressure from all areas of their environment, including themselves through the internalization of such norms. Similar to the literature, gay men within the present study emphasized both the direct and indirect nature of gender norm acquisition through direct interactions with others as well as through observations and imitation [2].

Participants within the present study also described instances of conformity to masculine norms, passing, and altering their public behavior/appearance. Behavior such as this is common among sexual minority individuals who experience fear and/or distress regarding both actual and anticipated discrimination and/or harassment. Gay men often expressed negative reactions to their public presentation. This suggests that individuals and society have a specific conception of how a gay man should outwardly appear/present. This example demonstrates not only that gayness has a physical appearance but also implies that one ought to avoid appearing this way. This derision of outward gayness highlights a particular notion prevalent within one’s society that, regardless of acceptance, homosexuality should remain in the closet. 

The constructs of gender and sexuality norms are described to be systemically and cyclically perpetuated through macro- and exo-level systems such as one’s society, culture, religion, traditions, educational institutions, and media. This is consistent with current theories that maintain one’s overarching social structures, systems, and institutions play a part in fostering and perpetuating such norms [38,39,41]. Additionally, individuals describe these norms as being regulated and enforced by agents within their meso- and micro-level systems, with whom they may have either direct or indirect contact. These agents include: mothers and fathers, relatives, friends, peers and acquaintances, individuals within one’s community, and even strangers. These results are consistent with other similar studies [4,25,29,30,31]. Additionally, the degree of pressure, regulation, and enforcement placed on the individual by these agents may vary. Phrases such as “that is so gay” are argued to be a form of social regulation to deter unscripted expressions of masculinity [10]. For instance, gay men in our study experienced conflicting and competing expectations from agents within their meso- and micro-systems. It was often described that gay men were expected to conform to masculine norms due to their identity as a man but were also expected to conform to feminine norms due their sexual identity. This often led to gay men experiencing a strain and conflict between their identities. This further maintains the notion of the term homosexual male being oxymoronic. Whether gay men adhere to one set of norms or the other, they risk ostracization due to one of their identities.

This paper suggests that there exists a strain experienced by gay men in relation to masculinity, in that it impedes on their relationship and forms of communicating with others within their micro- and meso-environments. Additionally, it argues that gay men experience a conflict between their identities as both a man and a gay man. Current heterosexist and heteronormative constructs of gender norms tend to dichotomize masculinity and homosexuality. As such, an oxymoron is created by the term gay man and arguably creates a strain uniquely experienced by gay men. This strain is described to impede on one’s relationships and their ability to actualize their potential. It is suggested that future research endeavor to examine this conflicting strain experienced by gay men and to examine whether it is an experience unique to gay men or shared amongst other non-heteronormative identities. Doing so will arguably aid in better understanding and accommodation in relieving gay men’s experiences of strain.

### Limitations and Recommendations

Some limitations of the study need to be pointed out here. Although the study only recruited participants identifying as gay men, we received interest from men identifying with other identities (e.g., bisexual). The present study is limited in that it only examines the effect of heteronormative gender norms on only one group (gay men). Future studies are recommended to examine other non-heteronormative identities in order to ascertain the broader spectrum of how gendered norms impact non-heteronormative individuals and whether these impacts are unique to particular sexual identities or are a shared experience. Specifically, there exists sparse research examining gender norms among bisexual men and little to no research utilizing trans and gender-diverse men [58]. Gender norms also impact heterosexual men in similar ways. Although there is a plethora of research on how conformity to masculine norms affect men’s health (see gender role conflict theory, gender role strain paradigm, and precarious manhood theory) and wellbeing, there is relatively limited research on non-conformity among straight men [42,59,60]. Future studies may wish to explore this avenue and/or employ a comparative study between heterosexual and LGBTI identities.

Additionally, the present study was limited in that the majority of participants identified as Caucasian and non-religious. Studies have revealed that men of diverse ethnicities and those high in religiosity are affected differently by gender and sexuality norms [61,62]. Future studies are recommended to examine a diverse sample consisting of different ethnicities and religious identification and to employ a comparative approach.

The year 2022 marks five years since the legalization of same-sex marriage within Australia through the Marriage Amendment (Definition and Religious Freedoms) Act 2017. The social environment within Australia, as well as globally, has changed over the years in regard to gender and sexual diversity [63,64]. The literature suggests that attitudes towards gender and sexual diversity improve following legislative change—specifically among those in support of it (i.e., LGBTI individuals, allies) [65]. Furthermore, online conferencing technologies have drastically improved as well through the increased use and accessibility of programs such as Zoom, including added features such as live closed-captioning, transcription, and breakout rooms [66,67]. As such, it is recommended that future studies examine heteronormative gender norms among LGBTI individuals (notably, from an Australian perspective) and to adopt a qualitative online conferencing approach. The increased usage and accessibility of such technologies will, arguably, support the implementation of this under-utilized methodology within the field and may also innovate new methodologies (e.g., online conference focus groups)

Many policies within Australia are informed by the determinants of the health model [68,69,70]. For example, the Australian Department of Health specifically highlights the importance of sex, gender, and socioeconomic characteristics (e.g., education, employment, income) as determinants of good health [71]. The socio-ecological model follows a similar structure and, by adopting both models, healthcare workers and researchers may ascertain specific areas that need to be addressed. The present study identified areas/sources of strain for gay men in each level of the socioecological environment. As such, the results aid policymakers map out sources of strain and allow for them to address them appropriately.

## 5. Conclusions

Sparse are current studies explicitly examining masculinity and internalized homonegativity [16,24,45], and even sparser are those employing the qualitative approach [13]. We addressed this gap by qualitatively examining Australian gay men’s perspectives on heteronormative gender norms (e.g., masculinity) and their experiences of being gay, identifying conflicts between their gender and sexual identities (i.e., gay man being an oxymoronic term), and the impact it has within varying socio-ecological systems. Our findings contribute to the furthering of the sociological understanding of LGBTI and men’s health and recommend future studies to further explore this topic in other LGBTI populations. Attitudes towards gender and sexuality diverse identities have also changed (notably following the legalization of same-sex marriage in Australia five years ago). As such, recommendations are made for future studies to replicate the study following the recent surge in usage of online conferencing technology.

## Figures and Tables

**Table 1:** Interview Question Guide.

Item No.	Question
1	How would you describe your understanding of society’s perceptions of male homosexuals.
2	It has been an old saying that gay men are typically feminine. What are your thoughts on this?
3a	What is your perception of what it means to be feminine?
3b	What is your perception of what it means to be masculine?
3c	Would you describe yourself as possessing more masculine/feminine characteristics?
4	Have you ever experienced pressure to behave more/less masculine/feminine?
5	Has this perception of homosexuality impacted your experiences growing up?
6	Have you ever experienced anti-feminine reactions from other people or been a witness to such an event?
7	Do you think it is important for men to act masculine?
8	I am about to read to you a few common feelings gay men have expressed in other studies about who they are.
8a	‘You can’t be a man and be gay’. What do you think of when you hear this?
8b	‘You’re less of a man simply because you don’t sleep with women’. What do you think of when you hear this?
8c	Have you ever felt or said anything like this before?
9	Do you ever have negative thoughts/feelings about being gay?
10	In your opinion, what influences gay men to feel negatively about their own queer identity?
11	This research hopes to reduce the stigmatization of what it means to be a gay man. Do you think reducing this stigma can help gay men experience less gender-related strain?

## Data Availability

The data associated with the paper are not publicly available but are available from the corresponding author upon reasonable request.
